# Transcriptome and functional analyses reveal *ERF053* from *Medicago falcata as* key regulator in drought resistances

**DOI:** 10.3389/fpls.2022.995754

**Published:** 2022-10-11

**Authors:** Qian Li, Wenbo Jiang, Zhihu Jiang, Wenxuan Du, Jiaxing Song, Zhiquan Qiang, Bo Zhang, Yongzhen Pang, Yuxiang Wang

**Affiliations:** ^1^Key Laboratory of Grassland Resources and Ecology of Western Arid Region, Ministry of Education, Key Laboratory of Grassland Resources and Ecology of Xinjiang, College of Grassland Science, Xinjiang Agricultural University, Urumqi, China; ^2^Institute of Animal Science, Chinese Academy of Agricultural Sciences, Beijing, China; ^3^College of Grassland Agriculture, Northwest A&F University, Shanxi, China

**Keywords:** *Medicago falcata*, Drought, RNA-Seq, WGCNA, *MfERF053*

## Abstract

*Medicago falcata* L. is an important legume forage grass with strong drought resistant, which could be utilized as an important gene pool in molecular breed of forage grass. In this study, *M. falcata* seedlings were treated with 400 mM mannitol to simulate drought stress, and the morphological and physiological changes were investigated, as well as the transcriptome changes of *M. falcata* seedlings at different treatment time points (0 h, 2 h, 6 h, 12 h, 24 h, 36 h and 48 h). Transcriptome analyses revealed four modules were closely related with drought response in *M. falcata* by WGCNA analysis, and four *ERF* transcription factor genes related with drought stress were identified (*MfERF053*, *MfERF9*, *MfERF034* and *MfRAP2.1*). Among them, *MfERF053* was highly expressed in roots, and MfERF053 protein showed transcriptional activation activity by transient expression in tobacco leaves. Overexpression of *MfERF053* in *Arabidopsis* improved root growth, number of lateral roots and fresh weight under drought, salt stress and exogenous ABA treatments. Transgenic *Arabidopsis* over-expressing *MfERF053* gene grew significantly better than the wild type under both drought stress and salt stress when grown in soil. Taken together, our strategy with transcriptome combined WGCNA analyses identified key transcription factor genes from *M. falcata*, and the selected *MfERF053* gene was verified to be able to enhance drought and salt resistance when over-expressed in *Arabidopsis*.

## Introduction

Drought is one of the most serious abiotic factors that can cause significant damage to both agriculture, human and livestock populations (Gupta et al., 2020). Forage grass are subjected to damage caused by drought stress due to the lack of rainfall and insufficient water supply over a long period of time, which eventually leads to a significant decrease in yield (Ray et al., 2015). Therefore, in order to largely prevent environmental damage, plants themselves have evolved specific regulatory protection mechanisms (Pinhero et al., 1997). During drought stress, dramatic changes occur from biochemical and physiological to gene expression and metabolic processes (Rao et al., 2020; Waititu et al., 2021). Drought stress system promotes the production of metabolites such as proline, initiates the antioxidant defense system internal to scavenge increased reactive oxygen species (ROS), prevents cell damage by scavenging free radicals, reduces the degree of membrane lipid peroxidation, and maintains membrane integrity (Wang et al., 2009; Anjum et al., 2011; Wei et al., 2019). Many drought related genes had been identified and used as candidate genes in genetic engineering, such as *EDT1* (Yu et al., 2016), *MfNACsa* (Duan et al., 2017), *CYT75B1* (Rao et al., 2020), *MYB30* (Wen et al., 2021), *CBF4* (Haake et al., 2002) and *ERF172* (Zhao et al., 2020). Among them, many of them are transcription factors, and they play key roles in regulating the expression of downstream targeted genes and metabolic pathway.

AP2/ERF superfamily transcription factors are one of the largest plant-specific transcriptional regulator groups in plants, with a conserved AP2/ERF DNA-binding structural domain of 57-66 amino acids in size (Okamuro et al., 1997). Ethylene responsive factors (ERFs) belong to AP2/ERF superfamily, which participate in plant response to hormone and abiotic stress (Qiang et al., 2010; Gibbs et al., 2015; Jung et al., 2017). In rice, overexpression of *JERF3* and *OsERF115*/*AP2*/*EREBP110* can increase the soluble sugar and proline content of transgenic plants, up-regulated the expression of *P5CS* gene under drought stress, and improve the tolerance of crops to drought and osmotic stress (Thoenes et al., 2004; Zhang and Huang, 2010). In addition, overexpression of tomato ethylene response factor ERF (TSRF1) in rice can improve permeability and drought resistance through binding GCC box, and up-regulated the expression of MYB, MYC, proline synthesis and photosynthesis-related genes (Quan et al., 2010), activated the expression of the abscisic acid (ABA) synthesis gene *SDR*, thereby enhanced the sensitivity of transgenic rice to ABA. However, overexpression of *OsDERF1* down-regulates ethylene synthesis and negatively affects drought tolerance (Zhai et al., 2012). *OsERF71* positively regulates ABA signaling to alter root structure and impart drought tolerance (Dong-Keun et al., 2016; Li et al., 2018). *NtERF172* acts as a positive factor in drought stress tolerance, transgenic tobacco showed higher oxidase activity, and lower H_2_O_2_ accumulation, in part by regulating the dynamic balance of CAT-mediated H_2_O_2,_ thereby exhibited greater drought tolerance (Zhao et al., 2020).

*M. falcata* is widely distributed in northern China, and most of them are wild resources with good drought resistance and good palatability, providing rich nutrients for cattle, sheep and other livestock (Yue and Zhou, 2004). By cross-pollination, *M. falcata* can be crossed with alfalfa to produce *Medicago varia* with stronger resistance and higher utilization value, therefore *M. falcata* is an important gene source for *Medicago* breeding (Wang et al., 2008; Kang et al., 2011). In *M. falcata*, some genes have been reported to be associated with abiotic stress response, including *MfNACsa* (Duan et al., 2017), *MfNAC3* (Qu et al., 2016), *MfUSP1* (Gou et al., 2020), and galactinol synthase gene 1 (*MfGolS1*) (Zhuo et al., 2013). At present, the functional study on *ERF* genes in response to drought are not clear in *M. falcata*.

In this study, we explored the physiological and molecular responses to drought stress in *M. falcata* seedlings, and the correlation analysis of the clustered modules with physiological indicators analyzed by WGCNA. Combined with these analysis, an *ERF* gene was found to be strongly correlated with drought-related module, suggesting a potential role in drought stress. Furthermore, we reported the functional characterization of *MfERF053* in conferring multiple resistances to abiotic stresses by over-expression in *Arabidopsis*.

## Materials and methods

### Plant materials and sample collection

Seeds of *M. falcata* were provided by the Key Laboratory of Grassland Resources and Ecology of Western Arid Region, Ministry of Education, College of Grass Industry, Xinjiang Agricultural University. The seedlings were grown at the Institute of Animal Science, Chinese Academy of Agricultural Sciences, Beijing. To ensure the consistency of seed germination, seeds with uniform size and fullness were selected and gently scratched with knife. The seeds were then sterilized with 75% ethanol for 10 min, 5% sodium hypochlorite for 10 min, followed by wash with sterile water for 4-5 times. The seeds were then sow on 1/2 MS medium, and placed at 4 °C for 3 days, and then in an light incubator at 22 °C for germination (16 h light/8 h darkness). Seedlings were transferred into flasks containing different concentrations of mannitol for drought treatment. The concentrations of mannitol were 200 mM, 300 mM, 400 mM, 500 mM, 600 mM, and the treatment without mannitol was set as the control group (CK) in this study.

In this experiment, the treatment with 400 mM mannitol were selected for physiological index determination, and seven different treatment time points were selected with 15-day-old seedlings, these samples were collected at 0 h (CK), 2 h, 6 h, 12 h, 24 h, 36 h and 48 h. For each treatment time point, three biological replicates were performed with 15 plants with whole seedlings for each replicate, samples were quickly collected and frozen in liquid nitrogen, and stored in a -80°C refrigerator.

### Measurement of physiological index

The content of MDA, Proline, SOD, CAT and POD in 15-day-old seedlings as for transcriptome sequencing were measured according to the instruction manual as provided at the website (https://www.solarbio.com/), and they were measured by using spectrophotometry methods. Statistical analysis was performed by one-way analysis of variance (ANOVA) and Duncan multiple tests using SPSS 22.0.

### RNA extraction and library construction for transcriptome analysis

The Eastep Super Total RNA Extraction Kit was used for RNA extraction (Promega Biotech, Shanghai, China). RNA quality was assessed on an Agilent 2100 Bioanalyzer (Agilent Technologies, Palo Alto, CA, USA) and checked using RNase free agarose gel electrophoresis. Twenty-one cDNA libraries were constructed, then the cDNA fragments were purified with QiaQuick PCR extraction kit (Qiagen, Venlo, The Netherlands), end repaired, poly(A) added, and ligated to Illumina sequencing adapters to sequence. The ligation products were size selected by agarose gel electrophoresis, PCR amplified, and sequenced using Illumina HiSeq2500 by Gene Denovo Biotechnology Co. (Guangzhou, China).

### Raw data processing, sequence assembly and functional annotation

Raw read containing adapters or low quality bases will affect the following assembly and analysis, which were uploaded in NCBI SRA (http://www.ncbi.nlm.nih.gov/sra): SRR19146603-SRR19146623. Thus, to get high quality clean reads, reads were further filtered by fastp (Chen et al., 2018) (version 0.18.0). An index of the reference genome was built (Chen et al., 2020), and paired-end clean reads were mapped to the reference genome using HISAT2. 2.4 (Kim et al., 2015) with “-rna-strandness RF” and other parameters set as a default.

### Identification of differentially expressed genes and PCA analysis

Analyses on differentially expressed genes were performed by DESeq2 software (Love et al., 2014) between two different groups, and by edgeR (Smyth, 2010) between two samples. The criteria of differentially expressed genes/transcripts (DEGs) screening was set as FDR<0.05 and |log_2_FC|>1. Principal component analysis (PCA) was performed with R package models (http://www.r-project.org/).

### WGCNA analysis

WGCNA (Weighted gene co-expression network analysis) is an analytical method to analyze the gene expression patterns of multiple samples (Zhang and Horvath, 2005), which allows clustering genes with similar expression patterns, and analyzing the association between modules and specific traits or phenotypes. Therefore, in this study, physiological indicator traits were analyzed in association with gene modules, using the R language package (Langfelder and Horvath, 2008). For annotation of the biological functions of the DEGs, GO and KEGG pathway enrichment analyses were performed with agriGO 2.0 (https://systemsbiology.cau.edu.cn/agriGOv2/) and KOBAS 3.0 (https://kobas.cbi.pku.edu.cn/), respectively.

### Quantitative real-time PCR

Four *MfERF* genes were selected for validation using qRT-PCR. *Actin* gene was amplified as internal standard gene, and AlleleID 6 Tool was used to design the gene-specific primers (**Table S1**), relative expression level was normalized by comparing with control and calculated using 2^-ΔΔCt^ method (Livak and Schmittgen, 2001), qRT-PCR analysis program was as follows: one cycle at 95°C for 15 min, followed by 40 cycles at 95°C for 10 s, 60°C for 20 s and 72°C for 30 s.

### Gene cloning and analyses on sequences and phylogenetic relationship

The full-length coding DNA sequence (CDS) of *MsERF053* was isolated from the roots of *Medicago falcata*, and cloned into pENTR vector for sequencing. The protein sequence of the homologous gene was selected by blast and multiple sequence comparisons. DNAMAN software was used to perform multiple sequence alignment. The phylogenetic tree was developed using protein sequences from *Medicago truncatula*, *Medicago sativa*, *Medicago ruthenica*, *Mucuna pruriens*, *Arabidopsis thaliana*, *Glycine sopa*, *Vigna angularis* and *Vigna radiata* with MEGA 7.0 (http://www.megasoftware.net) and visualize by using Evolview with bootstrap value of 1000 replications.

### Transactivation assay

The open reading frame of *MfERF053* was also cloned with gene-specific primers by seamless cloning with KOD polymerase. The transactivation construct was generated by inserting the full-length sequence of *MfERF053* into the *Kpn* I and *Xba* I sites of vector pCAMBIA1300BD. The BDGAL4 plasmid was used as control, and BDGAL4-MfERF053 recombinant plasmid were transformed into *A. tumefaciens* strain GV3101 and infiltrated into leaves of *N. benthamiana* as previously reported (Yang et al., 2021). After 48 h, the leave samples were taken separately, the proteins were extracted and the activity was determined.

### Phenotypic analysis of transgenic *Arabidopsis* over-expression *MfERF053*

*MfERF053* gene was cloned into the plant overexpression vector pB2GW7 and transformed into the *Agrobacterium tumefaciens* strain GV3101 for transformation in *Arabidopsis* by using floral dipping method. Three over-expression lines (OE19, OE20 and OE33) of T3 generation and wild type Columbia-0 (Col) plants were used for subsequent phenotype analyses. Seedlings were grown in soil at 24°C (16 h light/8 h darkness), 70%-80% relative humidity, and 400 μmol·m^-2^·s^-2^ light intensity.

We transferred four-day-old transgenetic and wild type *Arabidopsis* plants that were germinated on plates containing 1/2 MS medium to plates containing 1/2 MS medium supplied with different concentrations of mannitol (300 mM and 400 mM), NaCl (100 mM, 125 mM, 150 mM and 200 mM) and ABA (50 μM, 100 μM, 150 μM and 200 μM). Root length, lateral root number and fresh weight were measured after 10-day treatment. Each measurement contained 10 seedlings with triplicates. Seedlings grown in soil under normal conditions for 30 days were used for stress treatment. For drought stress, seedlings were grown without water for 15 days, then rewatered for 5 days. For salt stress, transgenic and wild type *Arabidopsis* plants were treated with 300 mM NaCl for 7 and 12 days, and the seedlings were photographed, respectively.

## Results

### Analysis of phenotypic and physiological indicators of *M. falcata* under drought stress

Initially, four-week-old *M. falcata* seedlings were subjected to mannitol treatment with concentration of 200 mM, 300 mM, 400 mM, 500 mM, and 600 mM. Mannitol treatment inhibited the growth of *M. falcata*, and the root length gradually decreased and the number of lateral roots significantly decreased with the increase of mannitol concentration (**Figure S1A**). These seedlings were more severely stressed and showed purplish-red root color and wilted leaves at mannitol concentration of 500 mM and 600 mM (**Figure S1A**), thus a relatively lower concentration of 400 mM mannitol was selected for subsequent experiments.

Seedlings were subjected to mannitol treatment (400 mM) at different time points (0 h, 2 h, 6 h, 12 h, 24 h, 36 h and 48 h). The seedlings grew normally at 2 h, and the leaves appear slightly wilted at 6 h, and started to lose water at 24 h, and finally the leaves wrinkled and severely wilted at 48 h (**Figure S1B**). It was clear that leaf wilting became more severe with stress time, and *M. falcata* responded to mannitol stress in a relatively short period of time.

**Figure 1:**
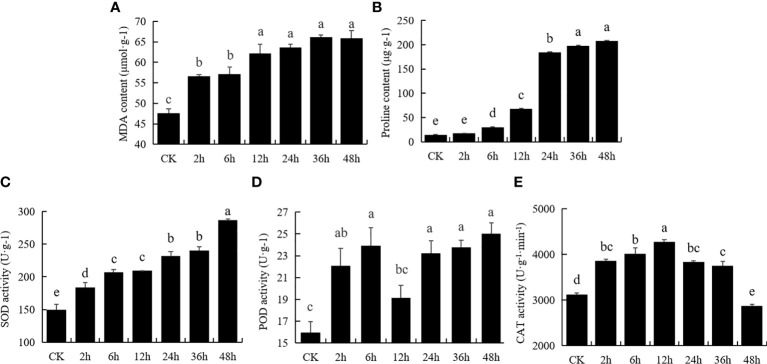
Physiological response of *Medicago falcata* to 400 mM mannitol treatment. Plant samples were collected under normal conditions and treated with 400 mM mannitol for 2 h, 6 h, 12 h, 24 h, 36 h and 48 h. The values are the average and error of three biological replicates. The same letters mean no significant difference, and different letters mean significant difference. **(A)**, MDA content; **(B)**, Proline content; **(C)**, SOD activity; **(D)**, POD activity; **(E)**, CAT activity.

To study the effect of drought stress on physiology changes in *M. falcata*, seedlings stressed with 400 mM mannitol at different time points were subjected to determine physiological indicators, including content of MDA, proline, and activity of SOD, POD and CAT (**Figure 1**). MDA content gradually increased from 2 h to about 55 μmol/g, and then gradually increased from 6 h to 48 h and reached the highest content at 48 h, indicating that the cells were most severely damaged by the treatment (**Figure 1A**). Drought stress significantly affected proline content in *M. falcata* plants at later stage (**Figure 1B**), with 10-fold increase (*p*<0.01) to 200 μg/g at 24 h, reaching maximum level at 48 h compared to untreated samples (**Figure 1C**). SOD activity of *M. falcata* plants increased with treatment time, began to increase significantly (*p*<0.05) after 2 h, and reached maximum level of approximately 280 U/g at 48 h, an increase of 186% compared to the control (**Figure 1C**). In terms of POD activity, the greatest increase was observed after 2 h treatment, with a slight increase at 6 h followed by a decrease at 12 h, with no significant differences from 24 h to 48 h (**Figure 1D**). CAT activity increased from 2 h to 36 h with its maximum value of 4200 U/g^-1^/min^-1^ at 12 h (**Figure 1E**). In conclusion, these physiological indicators of *M. falcata* responded to mannitol stress at different treatment time points with different degree.

### *De novo* transcriptome assembly and functional annotation of unigenes

The 21 cDNA libraries yielded 8,624,521,500 bp raw reads (**Table S2**), the clean data is 8,537,671,848 bp, the Q30 before and after filtration was relatively high (around 94%), and the GC content was around 44% (**Table S2**), indicating good sequencing quality. In order to assemble the sequencing data, the reference genome of alfalfa ecotype Xinjiang Daye was selected as the reference genome for comparison, and the sequencing results were assembled and annotated as shown in **Table S3**. For all samples, the unmapped reads were only about 6.92-10.78%, the unique mapped reads were 39.86-42.11%, and the total mapped reads accounted for 89.22-93.68%, thus these data clearly indicated that the genome sequences of *M. falcata* have a very high matching rate with that of *M. sativa*, which can be used for subsequent analysis.

### Identification and analysis of expression pattern of DEGs

Principal component analysis (PCA) showed that samples from CK were clustered into a separate category and they were separated away from the samples under stress treatments (**Figure S2**). Samples from 2 h and 6 h treatments were closer than with other treatments, samples from 12 h treatments were clustered into a separate category, and samples from 24 h, 36 h and 48 h with long duration stress treatments were clustered into one category (**Figure S2**). These data together indicated that samples from different treatments tend to cluster differently.

**Figure 2:**
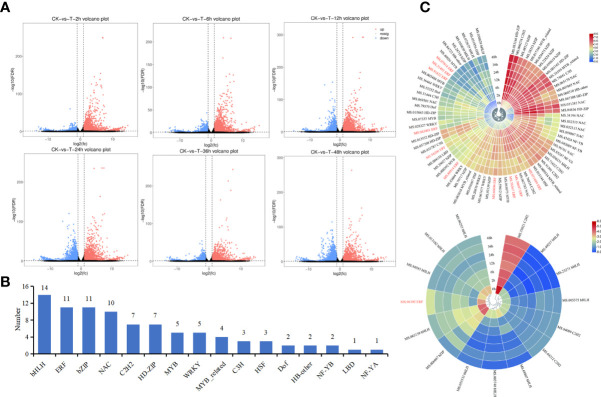
Analysis of differentially expressed genes at each time point under mannitol treatment. **(A)**, Volcano of difference among treatments. The horizontal coordinate indicates the log_2_(FC) of the difference between two groups, and the vertical coordinate indicates the negative log_10_ value of the FDR of the difference between the two groups. Red (up-regulated expression of group_2 relative to group_1) and blue (down-regulated expression) points indicate difference in gene expression (judged by FDR < 0.05, and more than two-fold difference), and black points indicate no difference. **(B)**, Statistic analysis of the number of different types of transcription factors. **(C)**, Heat map clustering of transcription factor expression. Red represents high expression and blue represents low expression. *MfERF* genes were highlighted in red.

The FPKM values for different samples were analyzed to investigate the changes in gene expression and to identify critical genes involved in drought stress in *M. falcata*. The volcano plot can be used to visualize the differentially expressed genes between treatment groups and control group. A total of 16,304 DEGs were obtained from transcriptome of *M. falcata* at 7 treatment time points. Among them, total 3,426, 3,632, 4,543, 3,944, 2,898, and 3,408 genes were down-regulated at 2 h, 6 h, 12 h, 24 h, 36 h and 48 h when compared with the control group at 0 h, respectively (**Figure 2A**). Meanwhile, 1,232, 2,165, 3,723, 4,156, 2,932, and 3,916 genes were down-regulated at 2 h, 6 h, 12 h, 24 h, 36 h and 48 h when compared with the control group at 0 h, respectively (**Figure 2A**).

Among these DEGs, 88 of them were found to be transcription factor genes belonging to 16 TF families (**Figure 2B**), including 14 *bHLH* genes, 11 *ERF* genes, 11 *bZIP* genes, and 10 *NAC* genes. Heat maps for the expression profiles of these 88 transcription factor genes under drought treatment showed that 73 of them were up-regulated compared with the control group (0 h) (**Figure 2C**, top), whereas 15 of them were down-regulated (**Figure 2C**, bottom). Among them, 10 out of 11 *ERF* genes were up-regulated (**Figure 2C**), indicating they may play leading roles in drought resistance in *M. falcata*.

### WGCNA of common DEGs in drought stress

**Figure 3:**
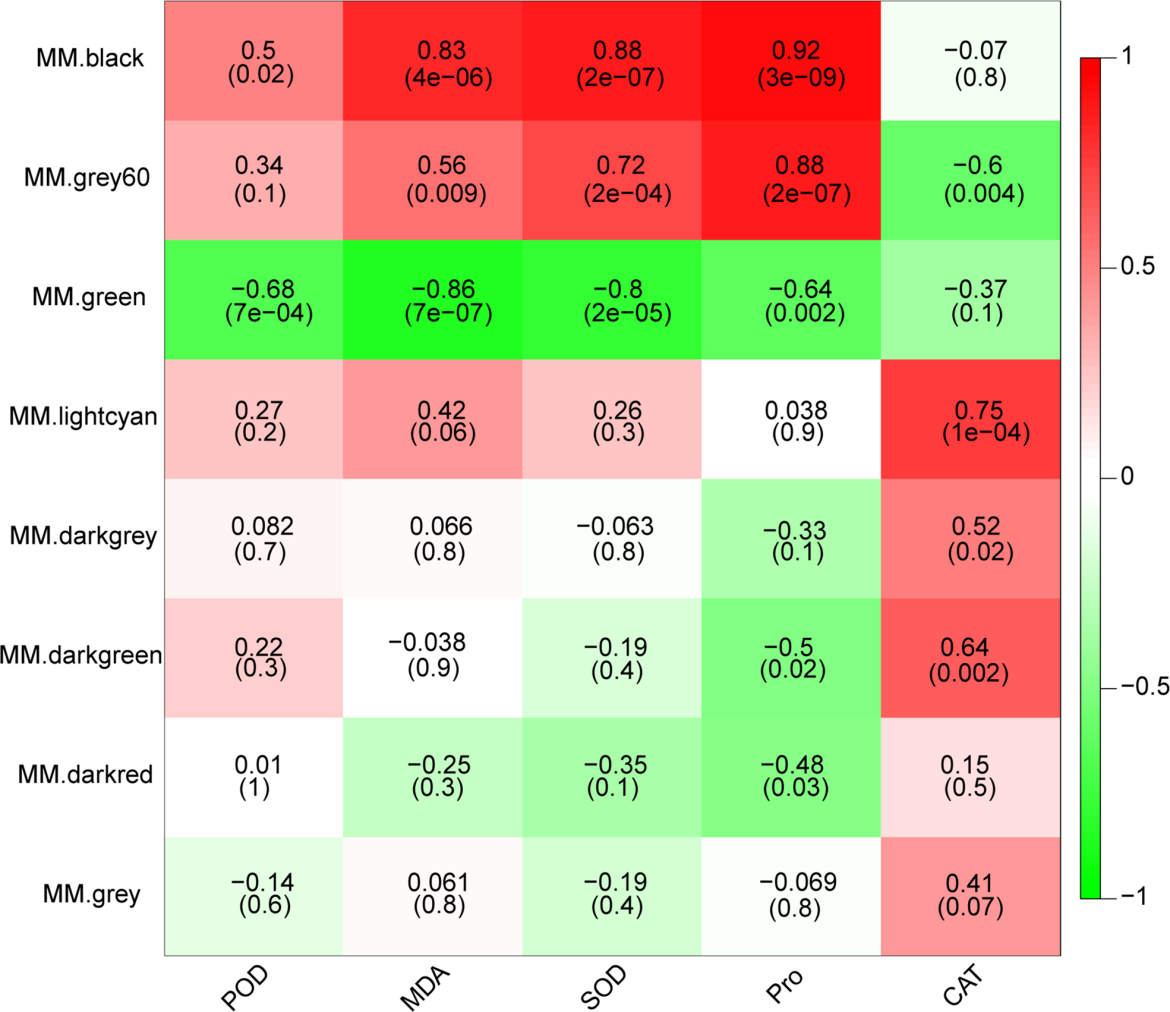
Character correlation diagram of physiological indicators. The horizontal coordinates indicate the character and the vertical coordinates indicate the module, which is plotted with Pearson correlation coefficient. Red represents positive correlation and green represents negative correlation. The darker the color, the stronger the correlation. The number in the brackets below represents significance *P*-value. The smaller the value, the stronger the significance.

In order to further investigate potential key genes involved in drought response in *M. falcata*, the weighted gene co-expression network was constructed using WGCNA, resulting in eight modules (**Figure S3**). In order to explore the correlation of these clustered modules with the above-mentioned physiological indicators, correlation analysis was performed between the module eigenvalues with activity of POD, CAT, SOD, content of Pro and MDA. Heat map was used to display the top correlation coefficient (**Figure 3**). The correlation coefficient between the black module and three indicators (MDA, SOD and Pro) were relatively high with values of 0.83, 0.88 and 0.92 (significance of 4e-06, 2e-07 and 3e-09), respectively (**Figure 3**). Grey60 module was significantly correlated with SOD activity and Pro content with correlations coefficient of 0.72 and 0.88, respectively (**Figure 3**). Both the lightcyan and darkgreen modules showed highly significant positive correlations with CAT activity, with correlation coefficients of 0.75 and 0.64 (significance of 1e-04 and 0.002), respectively (**Figure 3**). These data clearly indicated that four modules, namely black module, Grey60 module, lightcyan module and darkgreen module, were likely contain genes involved in drought responsive in *M. falcata.*

### Screening of candidate gene among DEGs

**Figure 4:**
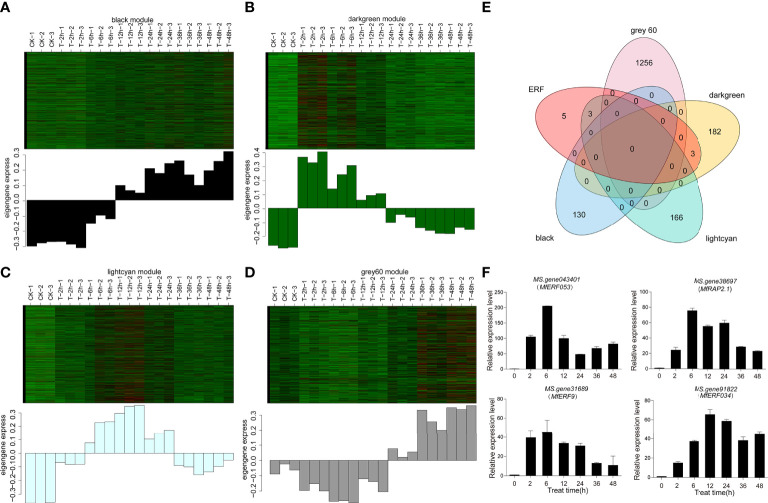
Differential candidate gene analysis. **(A–D)**, Heat map of gene expression pattern of each module. The above figure showed the expression level map of genes in modules in different samples; The following figure shows the characteristic values of modules in different samples. **(A–D)** represent darkgreen, lightcyan, black and gray60 module, respectively. **(E)**, The number of transcription factors differentially expressed in the five candidate combinations were screened by Venn map. Black, lightcyan, darkgreen and grey60 represent the three candidate modules screened by WGCNA, and *ERF* represents the number of *ERF* genes common shared by all treatments and controls. **(F)**, The expression level of four *MfERF* genes under mannitol treatment at different time point as detected by qRT-PCR.

The four modules (black, Grey60, lightcyan and darkgreen) were up-regulated modules, but they differed in expression patterns, which was consistent with the expected expression pattern of concern. The darkgreen module was up-regulated in early stage (2 h and 6 h) but down-regulated in the late stage (12 h, 36 h and 48 h, **Figure 4A**). The lightcyan module was up-regulated in the early and middle stage (2 h, 6 h, 12 h) and down-regulated in the late stage (36 h and 48 h, **Figure 4B**). The black and grey60 modules showed a higher expression in the late stage (36 h and 48 h) than the earlier time points (**Figure 4C, D**). Furthermore, venn diagram with these four modules and *ERF* genes showed that 6 *ERF* genes were expressed in two out of four modules (**Figure 4E**). Among these 6 genes, both *MS.gene31493* and *MS.gene043401* from the darkgreen modules were two different transcripts for the same gene *MfERF053*, and *MS.gene38367* and *MS.gene38697* were for *MfRAP2-1* gene (**Table S4**), therefore, the expression of these four genes, namely *MfERF053*, *MfERF9*, *MfRAP2-1* and *MfERF034* were further verified by qRT-PCR. It was revealed that the expression of these four genes were up-regulated to different levels under drought treatment at different time points (**Figure 4F**), which is consistent the transcriptome sequencing data as verified by correlation analyses (**Figure S4**). Among these four genes, *MfERF053* showed the highest expression level, which was thus selected for further investigation.

### Cloning, multiple sequence alignment and evolutionary tree analysis

**Figure 5:**
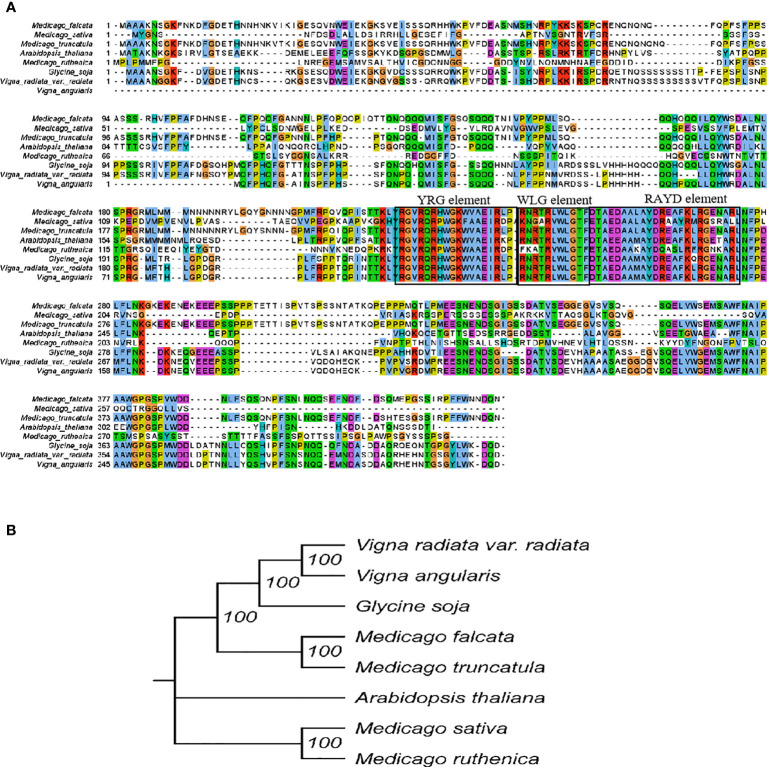
Sequence analysis of plant *ERF* genes. **(A)**, Multiple sequence alignment. The alignment were constructed by MEGA-X and visualized by Jalview. Residues with more than 50% similarity were shaded. Conserved regions (YRG element, WLG element and RAYD element) were indicated at the top. **(B)**, Phylogenetic analysis *ERF053* genes from different plant species.

The full-length open reading frame of *MfERF053* was cloned and the sequence was submitted to the National Center for Biotechnology Information (NCBI) under GenBank accession number of OM970125. Multiple sequence alignment of *MfERF053* with *ERF053* from other plant species showed that *MfERF053* shared 95%, 56%, 54% similarity with its homology genes from *M. truncatula*, *Vigna angularis* and *Glycine soja* at amino acid level (**Figure 5A**). All proteins from *M. truncatula*, *G. soja*, *V. angularis*, *Arabidopsis thaliana*, *Vigna radiata* var. *radiata*, *M. sativa*, *Medicago ruthenica* shared the characteristics features of DREB proteins, with conserved YRG, WLG, and RAYD motifs (**Figure 5A**).

Phylogenetic analyses revealed that *MfERF053* was clustered with *ERF053* from *M. truncatula* and *G. soja*, which was separated from those of *A. thaliana*, *M. sativa*, *M. ruthenica* with relatively low sequence similarity (51%, 49% and 48%, respectively, **Figure 5B**).

### Expression profile and transcriptional activity of MfERF053

**Figure 6:**
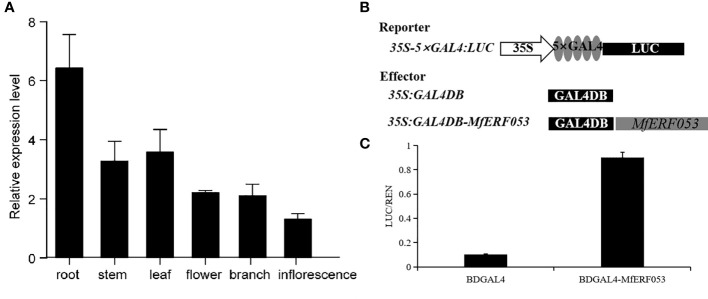
Characteristic of *MfERF053* gene. **(A)**, Validation of expression pattern of *M. falcata* in various tissues by qRT-PCR. **(B)**, Schematic diagram of reporter and effector for *MfERF053* gene for transactivation assay. **(C)**, *MfERF053* transcriptional activity analysis.

We analyzed relative expression level of *MfERF053* gene by qRT-PCR in roots, stems, leaves, flowers, branches and inflorescences of seedlings of *M. falacata* under normal growth condition. It was found that *MfERF053* was most highly expressed in roots, followed by in leaves and stems (**Figure 6A**). This finding suggests that *MfERF053* may be involved in mediating drought stress signaling through roots.

To investigate whether MfERF053 possesses transactivation activity, we generated a transactivation construct (35S::GAL4DB- MfERF053) and expressed it in tobacco epidermal cells by *Agrobacterium-*medicated transformation, using GAL4DB as a negative control (**Figure 6B**). It was revealed that MfERF053 could activate the GAL4-responsive expression of the LUC reporter protein, and the relative luciferase activity for MfERF053 were about 9 times higher than the control (GALDB), indicating that MfERF053 acts as a transcription activator (**Figure 6C**).

### Over-expression of *MfERF053* in *Arabidopsis* enhanced resistance under plate culture condition

**Figure 7:**
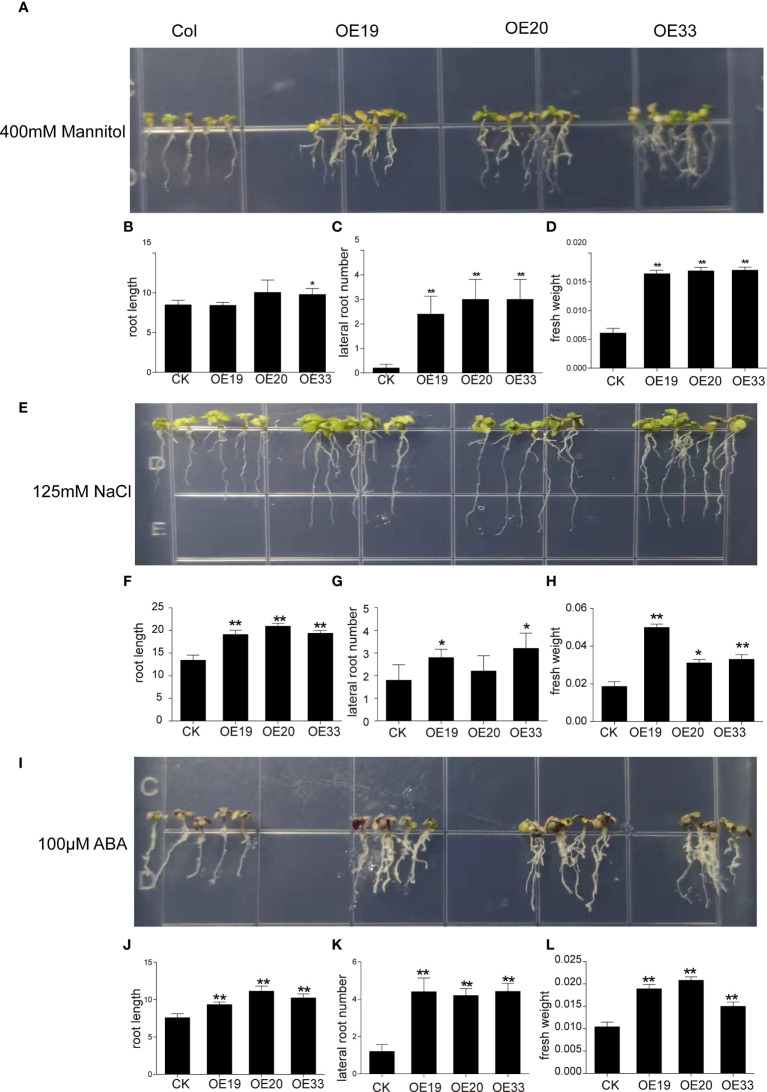
Evaluation of different resistance of *MfERF053* transgenic *Arabidopsis*. **(A, E, I)**, Plants overexpression *MfERF053* were treated with 400 mM mannitol, 100 mM NaCl and 100 μM ABA with wide type, respectively. **(B, F, J)**, Root length of different plant lines. **(C, G, K)**, Lateral root number of different plant lines. **(D, H, L)**, Fresh weight of different plant lines. Three replicates per treatment, 6 plants per replicate, * *p* < 0.05, ** *p* < 0.01, Duncan’s *t*-test.

qRT-PCRs were performed to measure the expression levels of *MfERF053* in leaves of the transgenic *Arabidopsis* of the T3 generation. Among twenty-five lines that were detected, three independent transgenic lines with relatively high expression level were selected for further analyses (lines OE19, OE 20, and OE 33). No significant difference were observed between the transgenic line and the wild type control under normal plate culture condition (**Figure S5**). Both the transgenic lines and the wild-type *Arabidopsis* seedlings were grown on plates supplied with mannitol of different concentrations (300 mM and 400 mM), NaCl (100 mM, 125 mM, 150 mM, and 200 mM) and ABA (50 μM, 100 μM, 150 μM and 200 μM) (**Figure 7**, **Figure S6**, **Figure S7**, **Figure S8**).

When treated with 300 mM mannitol and 400 mM mannitol for 10 days, the root length of the overexpression lines did not show any difference from the control (**Figure 7A**, **Figure S6**), but the number of lateral roots and fresh weight increased significantly compared with the control (*p* < 0.05). Moreover, the increase in both lateral root number and fresh weight under 400 mM mannitol treatment were greater than those with 300 mM mannitol treatment (**Figures 7B–D**, **Figure S6**).

After 10 days of treatment with 100 mM NaCl, overexpression lines grew significantly better than the wild type, showing longer root length and increased number of lateral roots (**Figure S7**). After 10 days of treatment with 125 mM NaCl stress, the chlorophyll content of the leaves in the wild-type plants decreased with yellow leaves, while the overexpression lines showed green leaves and increased in root length, number of lateral roots and fresh weight (**Figures 7E–H**). After 10 days of treatment with 150 mM NaCl stress, the wild-type plants turned white, but the overexpression lines remained green, with significantly more green leaves and fibrous root than the wild-type (**Figure S7**). After 10 days of treatment with 200 mM NaCl stress, both the wild-type and overexpression lines turned white, and their growth were severely inhibited, but leave color of the overexpression lines changed to light purple and some leaves turned white (**Figure S7**).

In addition, the wild-type and overexpression *Arabidopsis* were grown on plates supplied with different concentrations of ABA treatment for 10 d (Fig 7I, Fig S7). It was found that the leaves of the overexpression lines showed darker green and the number of lateral root increased after ABA stress with low concentration (50 and 100 μM) for 10 d (**Figure 7I**, **Figure S8**). However, high ABA concentration inhibited the growth of *Arabidopsis* roots, but the transgenic lines grew better than the wild-type (**Figure S8**). The root length, lateral root number and fresh weight of the overexpression lines were significantly increased compared with the wild type under ABA treatment (*p* < 0.01), indicating that overexpression of *MfERF053* gene could improve the sensitivity to ABA on root growth in *Arabidopsis* under certain concentration (**Figures 7J–L**, **Figure S8**).

### Over-expression of *MfERF053* in *Arabidopsis* enhanced resistance grown in soil

**Figure 8:**
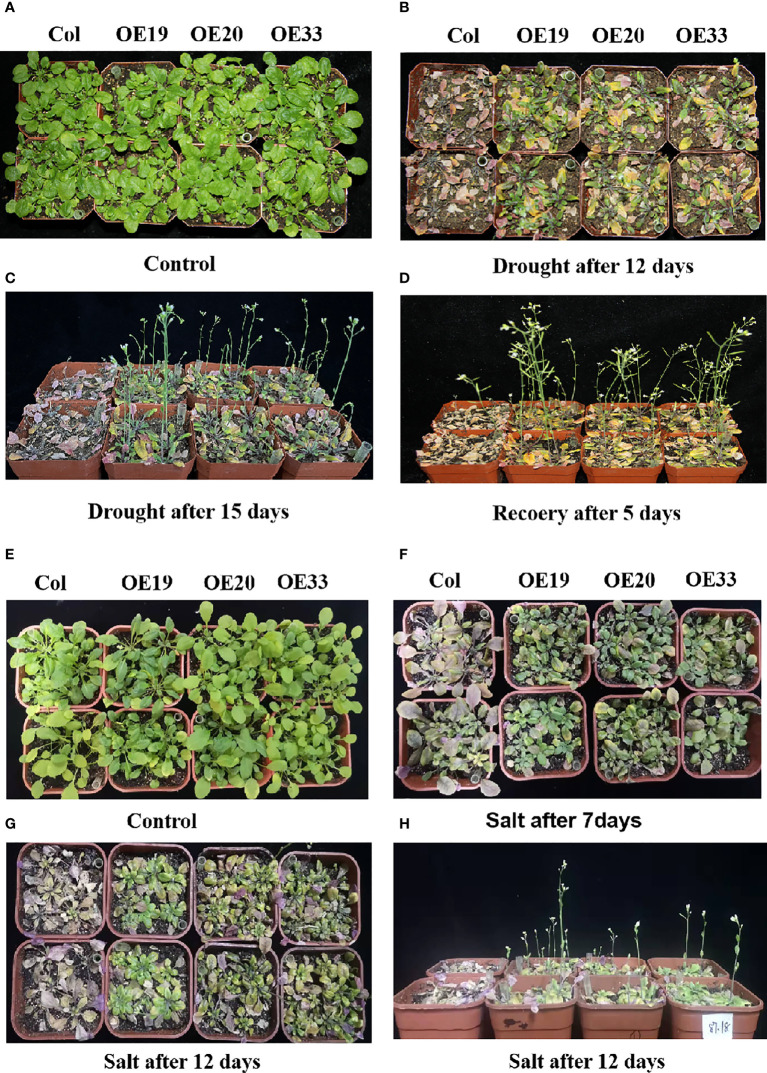
Phenotype of *MfERF053* transgenic overexpression *Arabidopsis* under drought NaCl and treatment in soil. **(A-D)**, The phenotype of wide type and transgenic of *Arabidopsis* under normal **(A)**, drought after 12 days **(B)**, drought after 15 days **(C)** and recover after 5 days **(D)**, respectively. **(E-H)**: The phenotype of wide type and transgenic of different strains of *Arabidopsis* under normal **(E)**, salt after 7 days **(F)**, salt after 12 days **(H)** and salt after 12 days **(I)**, respectively.

Three transgenic *Arabidopsis* strains (OE19, OE20 and OE33) showed no significant difference with the wild type control when plants grew in soil under natural watering condition (**Figure 8A**). But after 12 days of drought stress, wild-type *Arabidopsis* was more severely stressed than the transgenic lines (**Figure 8B**). The wild type plants turned significantly purple, whereas some of the older leaves of the transgenic lines were still green or green-yellow (**Figure 8B**). When drought stress was continued for 15 d, the wild-type *Arabidopsis* plants withered and could not grow, while the transgenic *Arabidopsis* continued to grow and flowered, and the leaves remained green (**Figure 8C**). After rehydration for 5 days, the wild-type *Arabidopsis* plants did not recovered and dried completely, while the overexpression *Arabidopsis* plants grew well and the leaves became green and the inflorescences could develop into pods normally, indicating that the transgenic *Arabidopsis* plants over-expressing *MfERF053* have conferred drought resistance (**Figure 8D**).

Both the transgenic and the wild type *Arabidopsis* were treated with 300 mM NaCl in the soil (**Figure 8E**). After 7 d of treatment, the leaves of the wild-type plants turned purple with significantly reduced chlorophyll, while the transgenic *Arabidopsis* remained green with few leaves turning purple (**Figure 8F**). After continuous stress for 12 days (**Figure 8G**), the wild-type *Arabidopsis* withered and died, while the transgenic *Arabidopsis* remained green with some old leaves turning purple, and they could still flower normally and grow (**Figure 8H**), indicating that the transgenic *Arabidopsis* conferred salt resistance when compared with the wild-type.

## Discussion

Among various environmental stress, drought is one of most serious stresses affecting the growth and development of plant. Drought stress triggers a series of responses from morphology to physiology, and to gene level. With the increase of drought stress, the antioxidant system of plants is destroyed, and the free radicals produced are greater than those cleared, resulting in excessive accumulation of ROS and membrane damage (Puyang et al., 2015; Nahar et al., 2017). In this study, the detailed information of physiological and transcriptome data of *M. falcata* under drought stress was provided. Under drought stress, the activities of SOD, POD and CAT increased (**Figure 1**), and the contents of proline and MDA increased, indicating that *M. falcata* has better ROS scavenging ability toward drought. It can be proven that the increase of these enzyme activities can eliminate stress-induced ROS and peroxides, inhibit plasma membrane peroxidation, and protect cells from damage (Zhang et al., 2004; Anjum et al., 2011; Koh et al., 2015; Quan et al., 2015; Xiong et al., 2022). The results of physiological indexes showed that *M. falcata* is capable of reducing the accumulation of harmful substances by regulating the activity of defense enzymes.

*M. falcata* is one of the candidate models to study abiotic stress response mechanism in legumes (Miao et al., 2015). The transcriptome analysis of drought provides a new biochemical and molecular mechanism for abiotic stress adaptation. Taking the genome of *M. sativa* cv ‘Xinjiang Daye’ as the reference genome, the tetraploid *M. falcata* transcriptome was sequenced, assembled and annotated, resulting in a total of 172,892 genes, with a comparison rate of more than 75% with the reference genome. The annotation of most genes is more similar to the annotation information of *M. truncatula*, indicating that the *M. falcata* transcriptome has good homologous sequence coverage, and it will also provide evidence for the expression of predicted genes in the genome of *M. truncatula* (Miao et al., 2015). Compared with the other transcriptome data for alfalfa with similar treatment (Luo et al., 2019), this study seems more complete. The number of genes obtained in each library in this study is higher than that produced in transcriptome of alfalfa under abiotic stress, which may be due to different samples used for sequencing. The sample for alfalfa is the roots, while the sample of this study is the whole seedlings. Therefore, the genes obtained for the transcriptome of alfalfa may be the genes specifically expressed in the roots, while in this study the obtained genes may be widely expressed in various parts.

In plants, some studies have screened stress resistance related genes through transcriptome sequencing (Gao et al., 2021; Li et al., 2022). In this study, we used *M. sativa* cv Xinjiang Daye as the reference genome, screened candidate genes through RNA-seq technology, and used expression patterns analysis and WGCNA analysis to mine hub *ERF* genes in response to dehydration of *M. falcata* (**Figures 3**, **4**). The biological functions of these genes need to be further explored and verified, but at least qRT-PCR for these four candidate genes were consistent with the transcriptome data, which proves the reliability of WGCNA co-expression network analysis method. In conclusion, this strategy of screening functional genes related to drought stress is of great significance to the study of stress resistance in *M. falcata* as in other studies (Qin et al., 2020).

A drought responsive *MfERF053* gene in *M. falcata* was screened in this study by transcriptome sequencing (**Figure 4**). Previous studies have reported that *ERF* transcription factors act as both activators and repressors of transcriptional functions (Yant et al., 2010). MfERF053 was shown to have transcriptional activation activity based on transcriptional activity assays (**Figure 6B**), which is similar as that of the *SlERF3* gene (Pan et al., 2010). The activation activity of MfERF053 is consistent with its lack of an EAR (ERF-associated amphiphilic repression) inhibitory element, therefore, it is reasonable that MfERF053 is involved in different biological processes mainly in the form of activator.

Compared with the wild type, overexpression lines with higher expression level of *MfERF053* showed significant changes in root length and lateral roots as well as fresh weight (**Figures 7**, **8**), indicating that overexpression of *MfERF053* had a significant effect on root growth in *Arabidopsis*. It has been shown that overexpression of the apple *MdERF11* and *MdERF106* genes (Han et al., 2020) can significantly improve the growth characteristics of plants to withstand abiotic stresses. Initial studies found that higher expression of *AtERF53* showed no significant difference in dehydration tolerance from wild type, it is speculated that the AtERF53 protein requires or may require post-translational modifications controlled by another mechanism (Hsieh et al., 2013). In addition, other studies showed that the E3 ligase RGLG1 E3 can promote the degradation of *PP2CA* through an ABA dependent pathway, and the RING E3 ligase *RGLG2* interacts with *AtERF53* to negatively regulate drought stress response in *Arabidopsis* (Cheng et al., 2012; Belda-Palazon et al., 2019). *GmERF113* can enhance the drought resistance through activating the expression of *PR10-1* by binding to the GCC-box in soybean plants (Fang et al., 2022). Overexpression of the *GmERF75* gene in soybean hairy roots showed stronger growth than wild type under 100 μmol/L^-1^ ABA and 120 mM NaCl treatment, indicating that overexpression of *GmERF75* improved soybean tolerance to salinity and exogenous ABA (Zhao et al., 2019). All these results suggested that *ERF* genes have a conserved role in response to abiotic stresses in different plant species. In this study, overexpression of *MfERF053* improved the resistance of *Arabidopsis* to osmotic stress through the ABA transduction pathway. Nevertheless, the regulation mechanism of *MfERF053* on drought and salt resistance in both *M. falcata* and in transgenic *Arabidopsis* requires further investigation in the near future.

## Data availability statement

The datasets presented in this study can be found in online repositories. The names of the repository/repositories and accession number(s) can be found in the article/**Supplementary Material**.

## Author contributions

QL and WJ designed this experiment, performed the experiments, and drafted the manuscript. ZJ, WD, JS, ZQ analyzed experimental data, visualized and perform experiment. BZ, YW and YP revised the manuscript and directed the study. All authors contributed to the article and approved the submitted version.

## Funding

This research was funded by National Nature Fund (31860675 and U1906201) and China Agriculture Research System of MOF and MARA (CARS-34).

## Conflict of interest

The authors declare that the research was conducted in the absence of any commercial or financial relationships that could be construed as a potential conflict of interest.

## Publisher’s note

All claims expressed in this article are solely those of the authors and do not necessarily represent those of their affiliated organizations, or those of the publisher, the editors and the reviewers. Any product that may be evaluated in this article, or claim that may be made by its manufacturer, is not guaranteed or endorsed by the publisher.

## Supplementary material

Supplementary Table 1 Primers used in this experiment.

Supplementary Table 2 Statistical information on transcriptome sequencing data.

Supplementary Table 3 Transcriptome alignment statistics.

Supplementary Table 4 Details of candidate genes.

Supplementary Figure 1 Phenotypic changes of *M. falcata* under mannitol treatment. **(A)**, Morphological response of *M. falcata* to different concentrations of mannitol. **(B)**, Effect of 400 mM mannitol drought stress at different times on leaf morphology of *M. falcata*.

Supplementary Figure 2 Principle component analysis of *M. falcata* at different times of 400 mM mannitol.

Supplementary Figure 3 Gene clustering map by WGCNA analysis. Left, Sample Hierarchical Clustering Tree. Right, Module level clustering diagram.

Supplementary Figure 4 Correlation analysis between qRNA-seq and RT-PCR data for the four candidate *MfERF* genes.

Supplementary Figure 5 Phenotype of *MfERF053* overexpression plants under normal plate growth condition. Col, the wide type control; Three over-expression lines: OE19, OE20 and OE33.

Supplementary Figure 6 Evaluation of overexpression plants *MfERF053* were treated with 300 mM mannitol. **(A)**, Overexpression of *Arabidopsis* 10-day phenotype under 300mM mannitol treatments. **(B)**, Root length of different plant lines. **(C)**, Lateral root number of different plant lines. **(D)**, Fresh weight of different plant lines.

Supplementary Figure 7 Evaluation of overexpression plants *MfERF053* were treated with different salt concentrations. **(A, E, I)**, Overexpression of *Arabidopsis* 10-day phenotype under 100 mM, 150 mM and 200 mM mannitol treatments, respectively. **(B, F, J)**, Root length of different plant lines. **(C, G, K)**, Lateral root number of different plant lines. **(D, H, L)**, Fresh weight of different plant lines.

Supplementary Figure 8 Evaluation of overexpression plants *MfERF053* were treated with different ABA concentrations. **(A, E, I)**, Overexpression of *Arabidopsis thaliana* 10-day phenotype under 100 μM, 150 μM and 200 μM ABA treatments, respectively. **(B, F, J)**, Root length of different plant lines. **(C, G, K)**, Lateral root number of different plant lines. **(D, H, L)**, Fresh weight of different plant lines.

## References

[B1] Anjum S. A. Wang L. Farooq M. Khan I. Xue L. (2011). Methyl jasmonate-induced alteration in lipid peroxidation, antioxidative defence system and yield in soybean under drought. J. Agron. Crop Sci. 197 (4), 296–301. doi: 10.1111/j.1439-037X.2011.00468.x

[B2] Belda-Palazon B. Julian J. Coego A. Wu Q. Zhang X. Batistic O.. (2019). ABA inhibits myristoylation and induces shuttling of the *RGLG1* E3 ligase to promote nuclear degradation of *PP2CA* . Plant J. 98, 813–825. doi: 10.1111/tpj.14274 30730075

[B3] Cheng M. C. Hsieh E. J. Chen J. H. Chen H. Y. Lin T. P. (2012). Arabidopsis RGLG2, functioning as a RING E3 ligase, interacts with *AtERF53* and negatively regulates the plant drought stress response. Plant Physiol. 158, 363–375. doi: 10.1104/pp.111.189738 22095047 PMC3252077

[B4] Chen H. T. Zeng Y. Yang Y. Z. Huang L. L. Tang B. L. Zhang H.. (2020). Allele-aware chromosome-level genome assembly and efficient transgene-free genome editing for the autotetraploid cultivated alfalfa. Nat. Commun. 11 (1), 2494–2505. doi: 10.1038/s41467-020-16338-x 32427850 PMC7237683

[B5] Chen S. F. Zhou Y. Q. Chen Y. R. Gu J. (2018). Fastp: an ultra-fast all-in-one FASTQ preprocessor. Bioinformatics 34 (17), 884–890. doi: 10.1093/bioinformatics/bty560 30423086 PMC6129281

[B6] Dong-Keun L. Suin Y. Youn S. Hyunyong K. (2016). Rice *OsERF71*-mediated root modification affects shoot drought tolerance. Plant Signal Behav. 12 (1), e1268311. doi: 10.1080/15592324.2016.1268311 PMC5289523 27935412

[B7] Duan M. Zhang R. Zhu F. Zhang Z. Gou L. Wen J.. (2017). A lipid-anchored NAC transcription factor is translocated into the nucleus and activates glyoxalase I expression during drought stress. Plant Cell 29 (7), 1748–1772. doi: 10.1105/tpc.17.00044 28684428 PMC5559744

[B8] Fang X. Ma J. Guo F. Qi D. Zhao M. Zhang C.. (2022). The AP2/ERF *GmERF113* positively regulates the drought response by activating GmPR10-1 in soybean. Int. J. Mol. Sci. 23 (15), 8159–8181. doi: 10.3390/ijms23158159 35897735 PMC9330420

[B9] Gao G. Lv Z. Zhang G. Li J. Zhang J. He C. (2021). An ABA-flavonoid relationship contributes to the differences in drought resistance between different sea buckthorn subspecies. Tree Physiol. 41 (5), 744–755. doi: 10.1093/treephys/tpaa155 33184668

[B10] Gibbs D. Conde J. V. Berckhan S. Mendiondo G. M. Prasad G. Holdsworth M. J. (2015). Group VII ethylene response factors coordinate oxygen and nitric oxide signal transduction and stress responses in plants. Plant Physiol. 169 (1), 23–31. doi: 10.1104/pp.15.00338 25944828 PMC4577381

[B11] Gou L. Zhuo C. Lu S. Guo Z. (2020). A universal stress protein from *Medicago falcata (MfUSP1)* confers multiple stress tolerance by regulating antioxidant defense and proline accumulation. Environ. Exp. Bot. 178, 104168–104179. doi: 10.1016/j.envexpbot.2020.104168

[B12] Gupta A. Rico-Medina A. Cao-Delgado A. I. (2020). The physiology of plant responses to drought. Science 368 (6488), 266–269. doi: 10.1126/science.aaz7614 32299946

[B13] Haake V. Cook D. Riechmann J. L. Pineda O. Thomashow M. F. Zhang J. Z. (2002). Transcription factor *CBF4* is a regulator of drought adaptation in arabidopsis. Plant Physiol. 130 (2), 639–648. doi: 10.1104/pp.006478 12376631 PMC166593

[B14] Han D. Han J. Yang G. Wang S. Xu T. Li W. (2020). An ERF transcription factor gene from *Malus* baccata l. borkh, *MbERF11*, affects cold and salt stress tolerance in *Arabidopsis* . Forests 11 (5), 514–529. doi: 10.3390/f11050514

[B15] Hsieh E. J. Cheng M. C. Lin T. P. (2013). Functional characterization of an abiotic stress-inducible transcription factor *AtERF53* in *Arabidopsis thaliana* . Plant Mol. Biol. 82 (3), 223–237. doi: 10.1007/s11103-013-0054-z 23625358

[B16] Jung H. Chung P. J. Park S. H. Redillas M. Kim Y. S. Suh J. W.. (2017). Overexpression of *OsERF48* causes regulation of *OsCML16*, a calmodulin-like protein gene that enhances root growth and drought tolerance. Plant Biotechnol. J. 15 (10), 1295–1308. doi: 10.1111/pbi.12716 28244201 PMC5595718

[B17] Kang Y. Han Y. Torres-Jerez I. Wang M. Tang Y. Monteros M.. (2011). System responses to long-term drought and re-watering of two contrasting alfalfa varieties. Plant J. 68 (5), 871–889. doi: 10.1111/j.1365-313X.2011.04738.x 21838776

[B18] Kim D. Landmead B. Salzberg S. L. (2015). HISAT: a fast spliced aligner with low memory requirements. Nat. Methods 12 (4), 357–U121. doi: 10.1038/Nmeth.3317 25751142 PMC4655817

[B19] Koh J. Chen G. Yoo M. J. Zhu N. Dufresne D. Erickson J. E.. (2015). Comparative proteomic analysis of *Brassica napus* in response to drought stress. J. Proteome Res. 14 (8), 3068–3081. doi: 10.1021/pr501323d 26086353

[B20] Langfelder P. Horvath S. (2008). WGCNA: an r package for weighted correlation network analysis. BMC Bioinf. 9 (1), 559. doi: 10.1186/1471-2105-9-559 PMC2631488 19114008

[B21] Li J. Guo X. Zhang M. Wang X. Zhao Y. Yin Z.. (2018). *OsERF71* confers drought tolerance *via* modulating ABA signaling and proline biosynthesis. Plant Sci. 270, 131–139. doi: 10.1016/j.plantsci.2018.01.017 29576066

[B22] Li P. Lin P. Zhao Z. Li Z. Liu Y. Huang C.. (2022). Gene co-expression analysis reveals transcriptome divergence between wild and cultivated sugarcane under drought stress. Int. J. Mol. Sci. 23 (1), 569–592. doi: 10.3390/ijms23010569 35008994 PMC8745624

[B23] Livak K. J. Schmittgen T. (2001). Analysis of relative gene expression data using real-time quantitative PCR and the 2-^△△Ct^ method. Methods 25 (4), 402–408. doi: 10.1006/meth.2001.1262 11846609

[B24] Love M. I. Huber W. Anders S. (2014). Moderated estimation of fold change and dispersion for RNA-seq data with DESeq2. Genome Biol. 15 (12), 550. doi: 10.1186/s13059-014-0550-8 25516281 PMC4302049

[B25] Luo D. Zhou Q. Wu Y. Chai X. Liu W. Wang Y.. (2019). Full-length transcript sequencing and comparative transcriptomic analysis to evaluate the contribution of osmotic and ionic stress components towards salinity tolerance in the roots of cultivated alfalfa (*Medicago sativa* L.). BMC Plant Biol. 19 (1), 32. doi: 10.1186/s12870-019-1630-4 30665358 PMC6341612

[B26] Miao Z. Xu W. Li D. Hu X. Liu J. Zhang R.. (2015). Denovo transcriptome analysis of *Medicago falcata* reveals novel insights about the mechanisms underlying abiotic stress-responsive pathway. BMC Genomics 16, 818. doi: 10.1186/s12864-015-2019-x 26481731 PMC4615886

[B27] Nahar K. Hasanuzzaman M. Alam M. M. Rahman A. Mahmud J. A. Suzuki T.. (2017). Insights into spermine-induced combined high temperature and drought tolerance in mung bean: osmoregulation and roles of antioxidant and glyoxalase system. Protoplasma 254 (1), 445–460. doi: 10.1007/s00709-016-0965-z 27032937

[B28] Okamuro J. K. Caster B. Villarroel R. Van M. M. Jofuku K. D. (1997). The AP2 domain of APETALA2 defines a large new family of DNA binding proteins in arabidopsis. PNAS 94 (13), 7076–7081. doi: 10.1073/pnas.94.13.7076 9192694 PMC21287

[B29] Pan I. C. Li C. W. Su R. C. Cheng C. P. Lin C. S. Chan M. T. (2010). Ectopic expression of an EAR motif deletion mutant of *SlERF3* enhances tolerance to salt stress and ralstonia solanacearum in tomato. Planta 232 (5), 1075–1086. doi: 10.1007/s00425-010-1235-5 20697739

[B30] Pinhero R. G. Rao M. V. Paliyath G. Murr D. P. Fletcher R. A. (1997). Changes in activities of antioxidant enzymes and their relationship to genetic and paclobutrazol-induced chilling tolerance of maize seedlings. Plant Physiol. 114 (2), 695–704. doi: 10.1104/pp.114.2.695 12223737 PMC158354

[B31] Puyang X. An M. Han L. Zhang X. (2015). Protective effect of spermidine on salt stress induced oxidative damage in two Kentucky bluegrass (*Poa pratensis *L.) cultivars. Ecotoxicol. Environ. Saf. 117, 96–106. doi: 10.1016/j.ecoenv.2015.03.023 25841065

[B32] Qiang Z. Zhang J. Gao X. Tong J. Xiao L. Li W.. (2010). The arabidopsis AP2/ERF transcription factor RAP2.6 participates in ABA, salt and osmotic stress responses. Gene 457 (1-2), 1–12. doi: 10.1016/j.gene.2010.02.011 20193749

[B33] Qin T. Y. Chao S. Zhen-Zhen B. Wen-Jun L. Peng-Cheng L. Jun-Lian Z.. (2020). Identification of drought-related co-expression modules and hub genes in potato roots based on WGCNA. Acta Agron. Sin. 46 (7), 19. doi: 10.3724/SP.J.1006.2020.94130

[B34] Quan R. Hu S. Zhang Z. Zhang H. Zhang Z. Huang R. (2010). Overexpression of an ERF transcription factor *TSRF1* improves rice drought tolerance. Plant Biotechnol. J. 8 (4), 476–488. doi: 10.1111/j.1467-7652.2009.00492.x 20233336

[B35] Quan W. Liu X. Wang H. Chan Z. (2015). Comparative physiological and transcriptional analyses of two contrasting drought tolerant alfalfa varieties. Front. Plant Sci. 6 1256. doi: 10.3389/fpls.2015.01256 26793226 PMC4709457

[B36] Qu Y. Duan M. Zhang Z. Dong J. Wang T. (2016). Overexpression of the *Medicago falcata* NAC transcription factor *MfNAC3* enhances cold tolerance in *Medicago truncatula* . Environ. Exp. Bot. 129, 67–76. doi: 10.1016/j.envexpbot.2015.12.012

[B37] Rao M. J. Xu Y. Tang X. Huang Y. Liu J. Deng X.. (2020). *CsCYT75B1*, a citrus CYTOCHROME P450 gene, is involved in accumulation of antioxidant flavonoids and induces drought tolerance in transgenic arabidopsis. Antioxid. (Basel) 9 (2), 161–182. doi: 10.3390/antiox9020161 PMC7070963 32079281

[B38] Ray I. M. Han Y. H., E, L. Meenach C. D. Santantonio N. Sledge M. K.. (2015). Identification of quantitative trait loci for alfalfa forage biomass productivity during drought stress. Crop Sci. 55 (5), 2012–2033. doi: 10.2135/cropsci2014.12.0840

[B39] Smyth G. K. (2010). edgeR: a bioconductor package for differential expression analysis of digital gene expression data. Bioinformatics 26 (1), 139. doi: 10.1093/bioinformatics/btp616 19910308 PMC2796818

[B40] Thoenes E. Dixit S. Pereira A. Aharoni A. Rooy G. V. Jetter R. (2004). The SHINE clade of AP2 domain transcription factors activates wax biosynthesis, alters cuticle properties, and confers drought tolerance when overexpressed in *Arabidopsis* . Plant Cell 16 (9), 2463–2480. doi: 10.1105/TPC.104.022897 15319479 PMC520946

[B41] Waititu J. K. Zhang X. Chen T. Zhang C. Zhao Y. Wang H. (2021). Transcriptome analysis of tolerant and susceptible maize genotypes reveals novel insights about the molecular mechanisms underlying drought responses in leaves. Int. J. Mol. Sci. 22 (13), 6980–7011. doi: 10.3390/ijms22136980 34209553 PMC8268334

[B42] Wang W. B. Kim Y. H. Lee H. S. Kim K. Y. Deng X. P. Kwak S. S. (2009). Analysis of antioxidant enzyme activity during germination of alfalfa under salt and drought stresses. Plant Physiol. Bioch. 47 (7), 570–577. doi: 10.1016/j.plaphy.2009.02.009 19318268

[B43] Wang J. J. Yun J. F. Shi-Jie L. V. (2008). Characteristic and utilizing values on the germplasm of *Medicago falcata* . J. Inner Mongolia Agric. Univ. (Natural Sci. Edition) 29 (1), 216–220. doi: 10.3901/JME.2008.09.177

[B44] Wei T. Wang Y. Xie Z. Guo D. Chen C. Fan Q.. (2019). Enhanced ROS scavenging and sugar accumulation contribute to drought tolerance of naturally occurring autotetraploids in poncirus trifoliata. Plant Biotechnol. J. 17 (7), 1394–1407. doi: 10.1111/pbi.13064 30578709 PMC6576089

[B45] Wen X. Geng F. Cheng Y. Wang J. (2021). Ectopic expression of *CsMYB30* from citrus sinensis enhances salt and drought tolerance by regulating wax synthesis in *Arabidopsis thaliana* . Plant Physiol. Biochem. 166, 777–788. doi: 10.1016/j.plaphy.2021.06.045 34217134

[B46] Xiong Y. Fan X. H. Wang Q. Yin Z. G. Sheng X. W. Chen J.. (2022). Genomic analysis of soybean PP2A-b family and its effects on drought and salt tolerance. Front. Plant Sci. 2 (12), 784038. doi: 10.3389/fpls.2021.784038 PMC8847135 35195114

[B47] Yang J. Li Q. Du W. Yao Y. Shen G. Jiang W.. (2021). Genome-wide analysis of glycoside hydrolase family 35 genes and their potential roles in cell wall development in *Medicago truncatula* . Plants (Basel) 10 (8), 1639–1655. doi: 10.3390/plants10081639 34451684 PMC8401519

[B48] Yant L. Mathieu J. Dinh T. T. Ott F. Lanz C. Wollmann H.. (2010). Orchestration of the floral transition and floral development in arabidopsis by the bifunctional transcription factor APETALA2. Plant Cell 22 (7), 2156–2170. doi: 10.1105/tpc.110.075606 20675573 PMC2929098

[B49] Yue X. Q. Zhou D. W. (2004). Good characteristics and its utilization of *Medicago falcata* . Jilin J. Anim. Husb. Vet. Med. 8, 26–28. doi: 10.3969/j.issn.1672-2078.2004.08.011

[B50] Yu L. H. Wu S. J. Peng Y. S. Liu R. N. Chen X. Zhao P.. (2016). *Arabidopsis EDT1/HDG11* improves drought and salt tolerance in cotton and poplar and increases cotton yield in the field. Plant Biotechnol. J. 14 (1), 72–84. doi: 10.1111/pbi.12358 25879154 PMC11389178

[B51] Zhai Y. Li J. W. Li X. W. Lei T. T. Wang Q. Y. (2012). Isolation and characterization of a novel transcriptional repressor *GmERF6* from soybean. Chem. Eng. J. 57 (1), 624–629. doi: 10.1007/s10535-012-0146-7

[B52] Zhang B. Horvath S. (2005). A general framework for weighted gene co-expression network analysis. Stat. Appl. Genet. Mol. 4 (1), 1–40. doi: 10.2202/1544-6115-1128 16646834

[B53] Zhang Z. Huang R. (2010). Enhanced tolerance to freezing in tobacco and tomato overexpressing transcription factor *TERF2/LeERF2* is modulated by ethylene biosynthesis. Plant Mol. Biol. 73 (3), 241–249. doi: 10.1007/s11103-010-9609-4 20135196

[B54] Zhang H. Huang Z. Xie B. Chen Q. Tian X. Zhang X.. (2004). The ethylene, jasmonate, abscisic acid and NaCl-responsive tomato transcription factor *JERF1* modulates expression of GCC box-containing genes and salt tolerance in tobacco. Planta 220 (2), 262–270. doi: 10.1007/s00425-004-1347-x 15300440

[B55] Zhao Q. Hu R. S. Liu D. Liu X. Wang J. Xiang X. H.. (2020). The AP2 transcription factor *NtERF172* confers drought resistance by modifying *NtCAT* . Plant Biotechnol. J. 18 (12), 2444–2455. doi: 10.1111/pbi.13419 32445603 PMC7680539

[B56] Zhao M. J. Yin L. J. Liu Y. Ma J. Zheng J. C. Lan J. H.. (2019). The ABA-induced soybean ERF transcription factor gene *GmERF75* plays a role in enhancing osmotic stress tolerance in *Arabidopsis* and soybean. BMC Plant Biol. 19 (1), 506. doi: 10.1186/s12870-019-2066-6 31747904 PMC6865046

[B57] Zhuo C. L. Wang T. Lu S. Y. Zhao Y. Q. Li X. G. Guo Z. F. (2013). A cold responsive galactinol synthase gene from *Medicago falcata (MfGolS1)* is induced by myo-inositol and confers multiple tolerances to abiotic stresses. Physiol. Plantarum 149, 1, 67–78. doi: 10.1111/ppl.12019 23253102

