# Supplementary material for: Transcriptome and functional analyses reveal ERF053 from Medicago falcata as key regulator in drought resistances
Source: Front Plant Sci. 2022 Oct 11;13:995754. doi: 10.3389/fpls.2022.995754 (PMC9594990; doi:10.3389/fpls.2022.995754)
Supplement: Supplementary Figure 1 — Phenotypic changes of M. falcata under mannitol treatment. (A), Morphological response of M. falcata to different concentrations of mannitol. (B), Effect of 400 mM mannitol drought stress at different times on leaf morphology of M. falcata. [file DataSheet_1.docx]

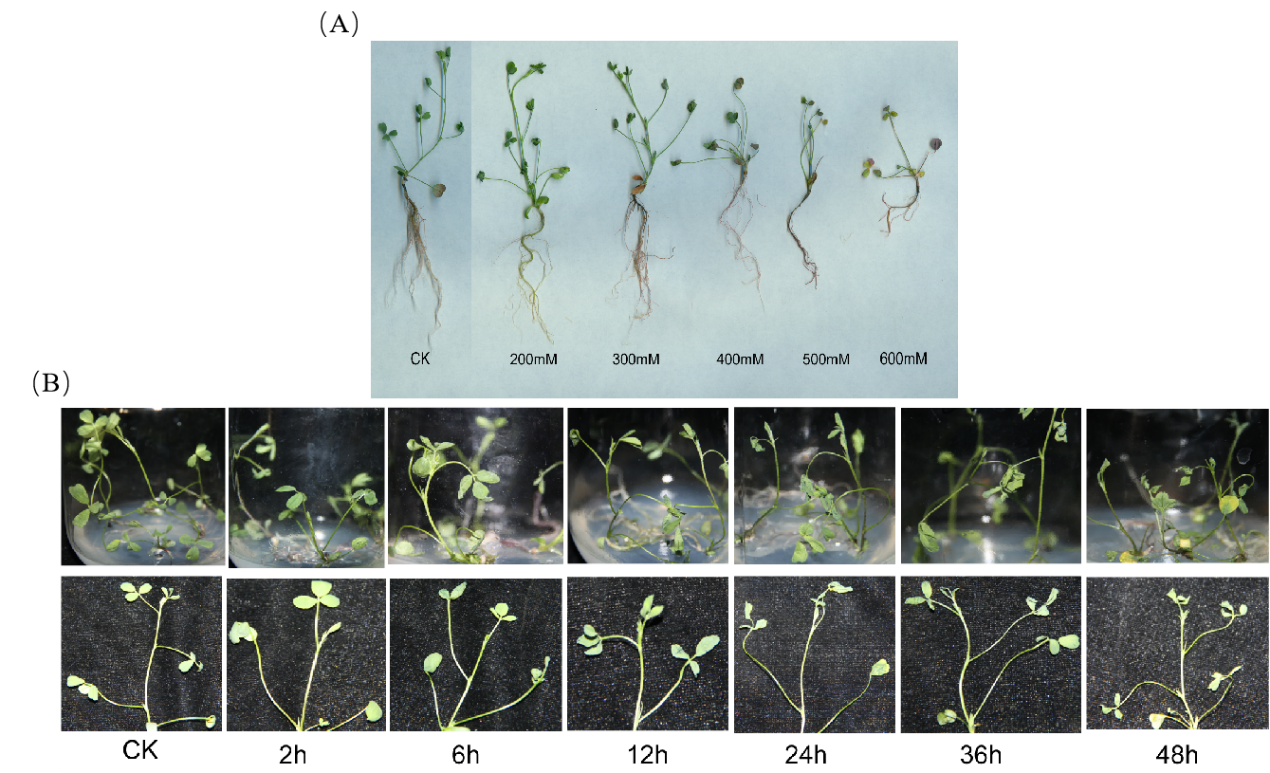


**Fig. S1.** Phenotypic changes of *M. falcata* under mannitol treatment. (A): Morphological response of *Medicago falcata* to different concentrations of mannitol. (B): Effect of 400 mM mannitol drought stress at different times on leaf morphology of *M. falcata*.


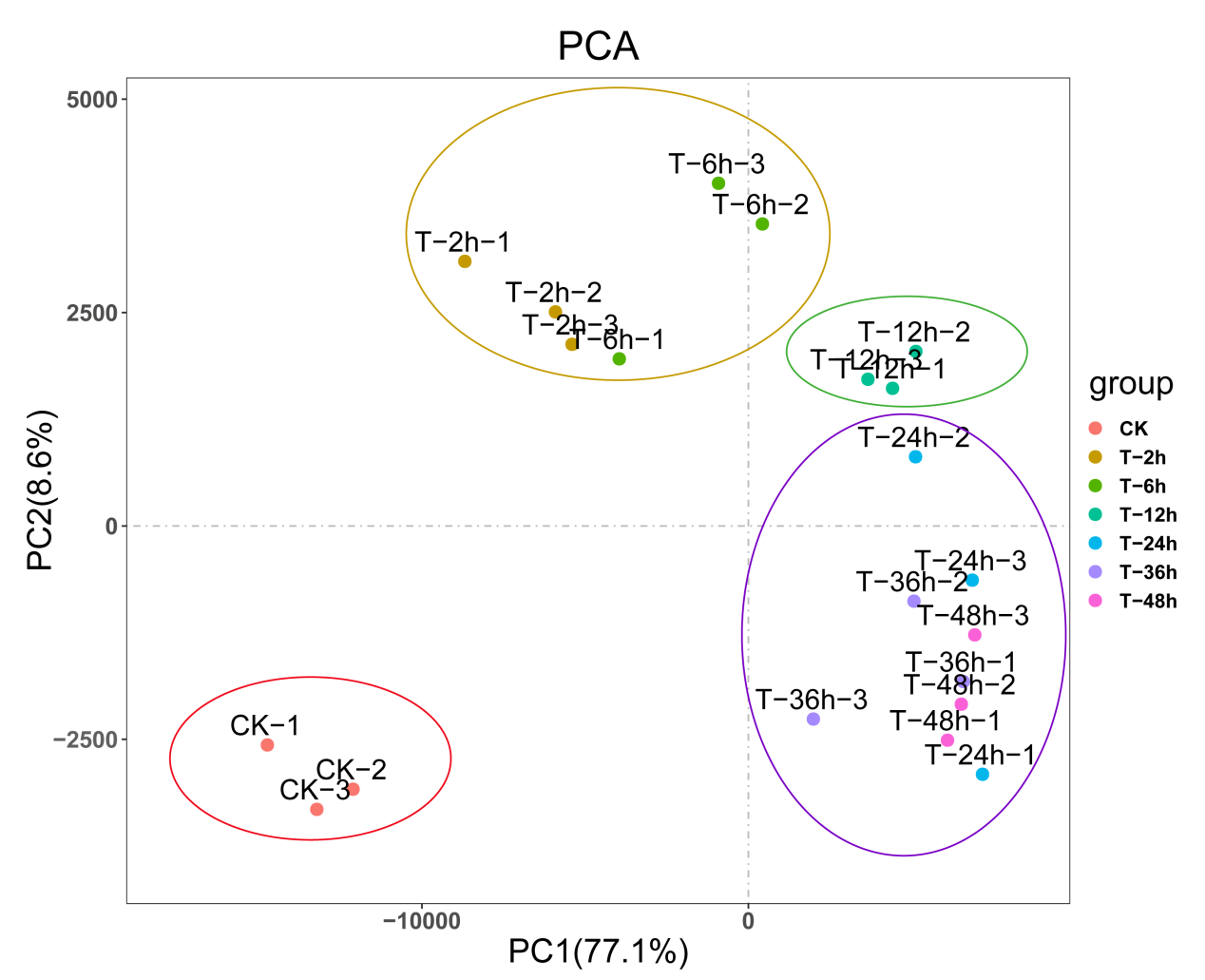


**Fig. S2.** Principle component analysis of *Medicago falcata* at different times of 400 mM mannitol


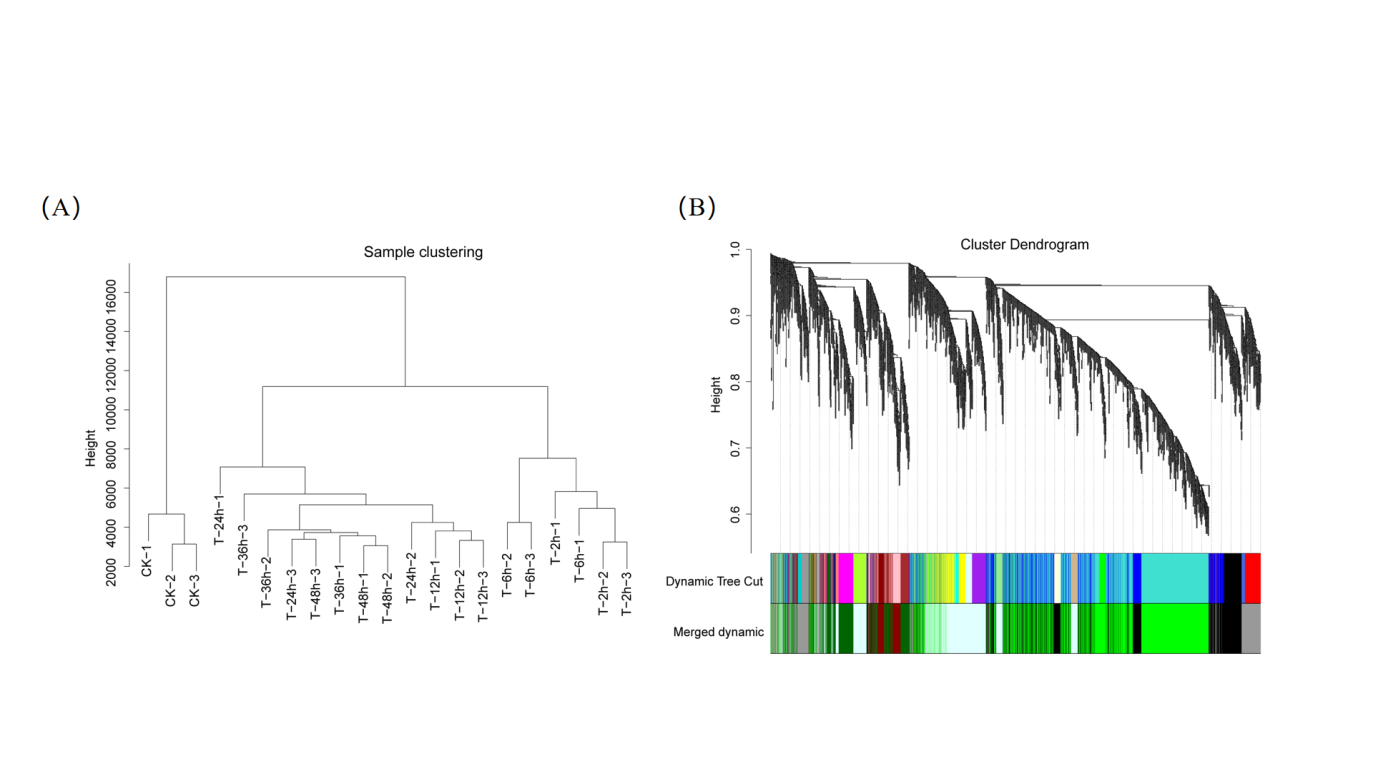


**Fig. S3.** Gene clustering map by WGCNA analysis. Left: Sample Hierarchical Clustering Tree. Right: Module level clustering diagram.


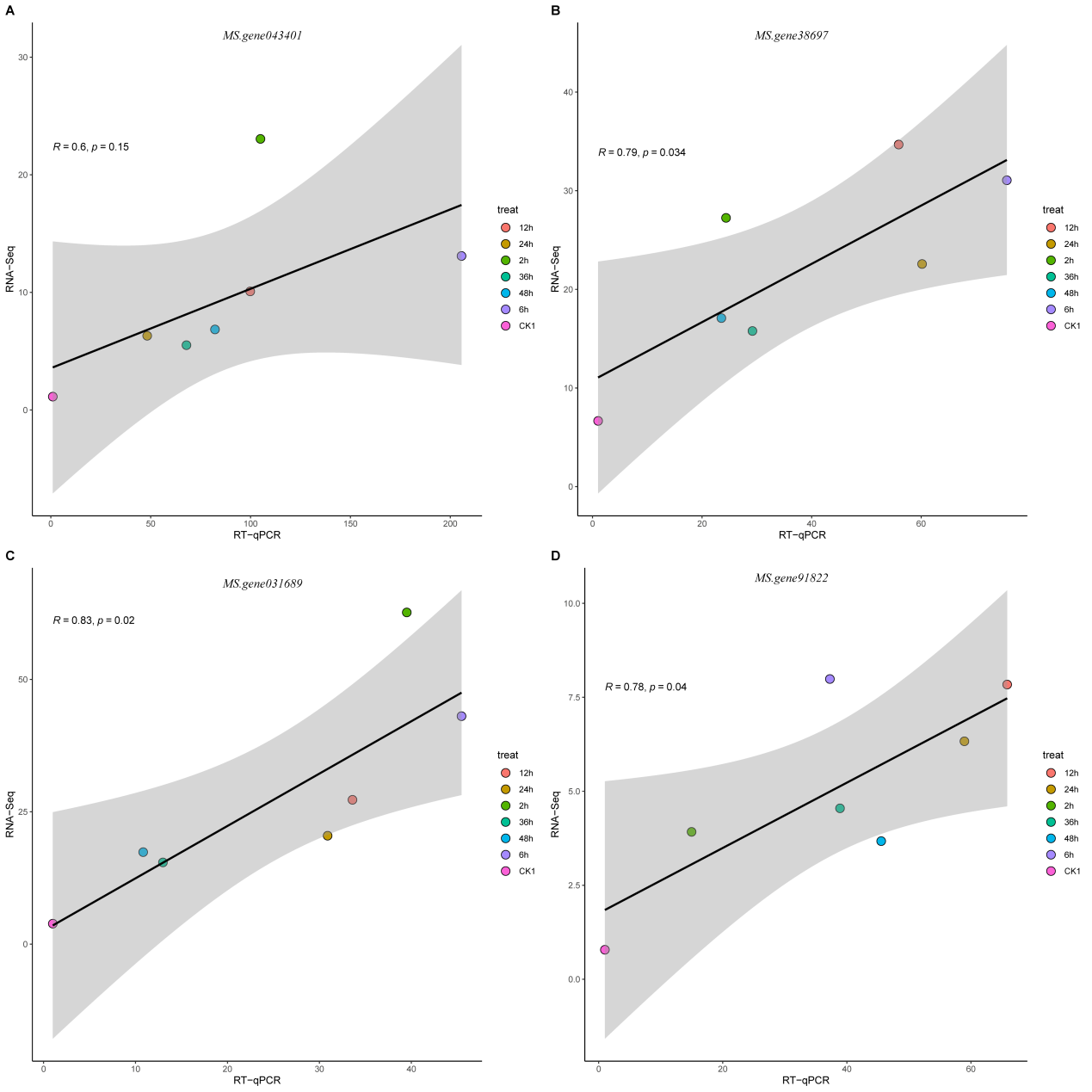


**Fig. S4.** Correlation analysis between RNA-seq and RT-qPCR data for the four candidate *MfERF* genes.


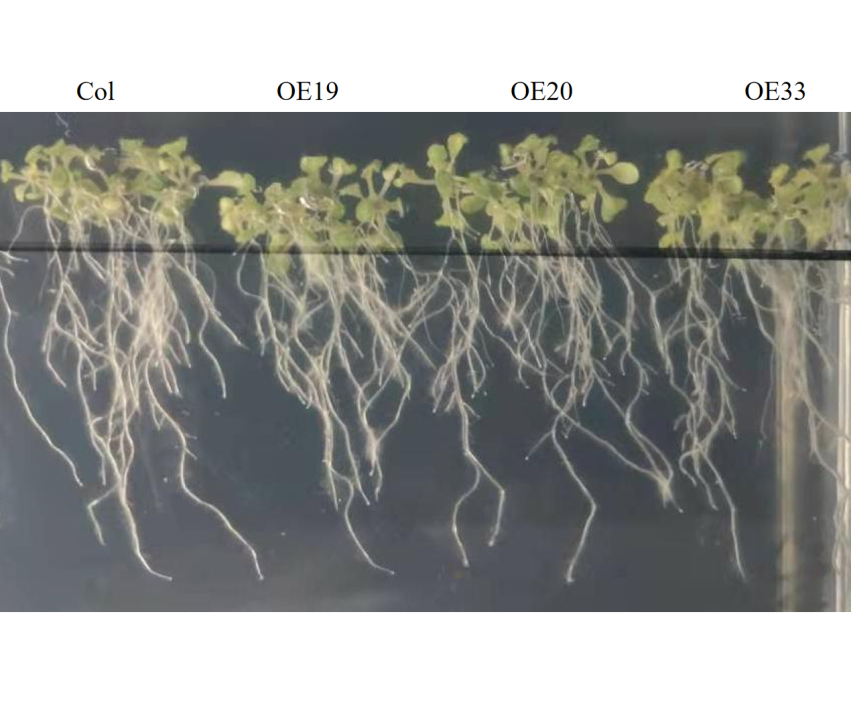


**Fig. S5.** Phenotype of *MfERF053* overexpression plants under normal plate growth condition. Col, the wide type control; Three over-expression lines: OE19, OE20 and OE33


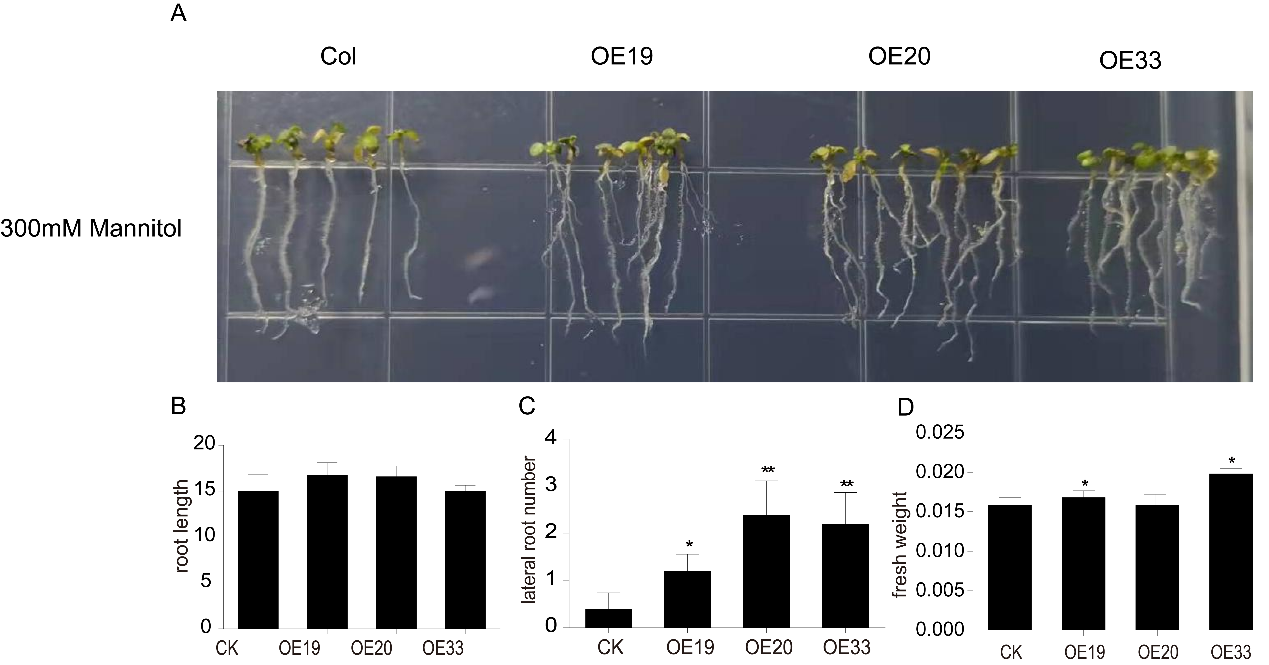


**Fig. S6.** Evaluation of overexpression plants *MfERF053* were treated with 300 mM mannitol. (A): Overexpression of *Arabidopsis* 10-day phenotype under 300mM mannitol treatments. (B): Root length of different plant lines. (C): Lateral root number of different plant lines. (D): Fresh weight of different plant lines.


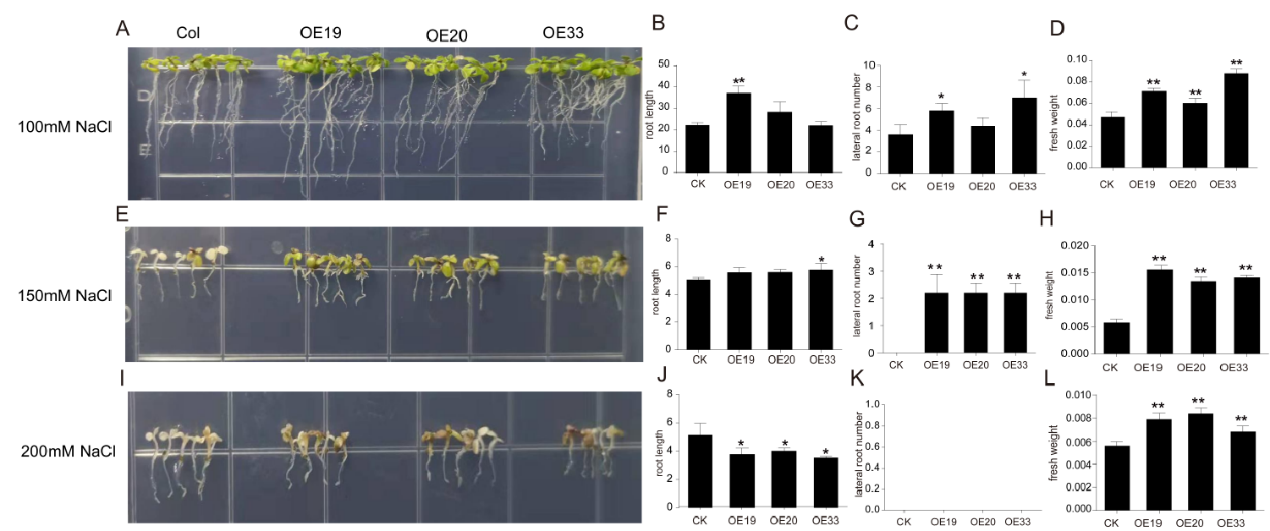


**Fig. S7.** Evaluation of overexpression plants *MfERF053* were treated with different salt concentrations (A, E, I): Overexpression of *Arabidopsis* 10-day phenotype under 100mM, 150mM and 200mM mannitol treatments, respectively. (B, F, J): Root length of different plant lines. (C, G, K): Lateral root number of different plant lines. (D, H, L): Fresh weight of different plant lines.


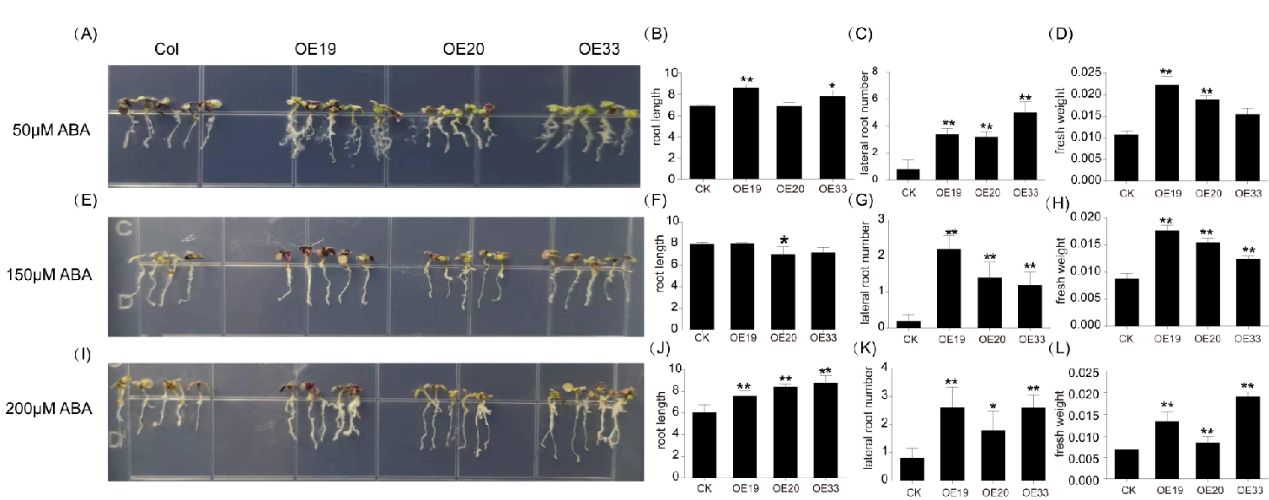


**Fig. S8.** Evaluation of overexpression plants *MfERF053* were treated with different ABA concentrations (A, E, I): Overexpression of *Arabidopsis thaliana* 10-day phenotype under 100μM, 150μM and 200μM ABA treatments, respectively. (B, F, J): Root length of different plant lines. (C, G, K): Lateral root number of different plant lines. (D, H, L): Fresh weight of different plant lines.
